# Bovine Parainfluenza-3 Virus Detection Methods and Prevalence in Cattle: A Systematic Review and Meta-Analysis

**DOI:** 10.3390/ani14030494

**Published:** 2024-02-02

**Authors:** Gebremeskel Mamu Werid, Thien D. Van, Darren Miller, Farhid Hemmatzadeh, Robert W. Fulton, Roy Kirkwood, Kiro Petrovski

**Affiliations:** 1 Davies Livestock Research Centre, School of Animal & Veterinary Sciences, University of Adelaide, Roseworthy Campus, Roseworthy, SA 5371, Australia; 2 Australian Centre for Antimicrobial Resistance Ecology, School of Animal & Veterinary Sciences, University of Adelaide, Roseworthy Campus, Roseworthy, SA 5371, Australia; 3 Department of Veterinary Pathobiology, College of Veterinary Medicine, Oklahoma State University, Broken Arrow, OK 74014, USA; 4 School of Animal & Veterinary Sciences, University of Adelaide, Roseworthy Campus, Roseworthy, SA 5371, Australia

**Keywords:** bovine respiratory disease complex, bovine parainfluenza virus type 3, antibody, antigen, nucleic acid, virus isolation

## Abstract

**Simple Summary:**

This study presents a comprehensive analysis of the global prevalence of bovine parainfluenza-3 virus (BPI3V) in cattle, a significant pathogen in the bovine respiratory disease complex (BRDC). By systematically reviewing and meta-analysing data from 71 articles across 32 countries, the study reveals variations in BPI3V prevalence based on different detection methods, including antibodies, antigen and nucleic acid detection, and virus isolation. The findings indicate a similar prevalence of BPI3V in cattle with BRDC compared to that in the general cattle population. This research underscores the need for dedicated BPI3V studies to better understand its role within BRDC and inform targeted disease management strategies.

**Abstract:**

Bovine parainfluenza-3 virus (BPI3V) is an important respiratory pathogen in cattle, contributing to syndromes in the bovine respiratory disease complex (BRDC). Despite its significance, the understanding of its prevalence remains fragmented, especially within the larger framework of BRDC. This systematic review and meta-analysis aimed to determine the global prevalence of BPI3V in cattle using varied detection methods and to highlight associated risk factors. Of 2187 initially retrieved articles, 71 were selected for analysis, covering 32 countries. Depending on the detection method employed, the meta-analysis revealed significant variations in BPI3V prevalence. In the general cattle population, the highest prevalence was observed using the antibody detection method, with a proportion of 0.64. In contrast, in cattle with BRDC, a prevalence of 0.75 was observed. For the antigen detection method, a prevalence of 0.15 was observed, exclusively in cattle with BRDC. In nucleic acid detection, a prevalence of 0.05 or 0.10 was observed in the general and BRDC cattle populations, respectively. In virus isolation methods, a prevalence of 0.05 or 0.04 was observed in the general and BRDC cattle populations, respectively. These findings highlight the differences in the detection ability of different methods in identifying BPI3V. Other factors, such as country, study year, coinfections, farm size, the presence of respiratory signs, sex, and body weight, may also affect the prevalence. Most studies were anchored within broader BRDC investigations or aimed at detecting other diseases, indicating a potential under-representation of focused BPI3V research. BPI3V plays an important role in BRDC, with its prevalence varying significantly based on the detection methodology. To further understand its unique role within BRDC and pave the way for targeted interventions, there is an evident need for independent, dedicated research on BPI3V.

## 1. Introduction

Bovine parainfluenza-3 virus (BPI3V) is a respiratory pathogen that predominantly affects cattle globally [1,2,3]. BPI3V can lead to secondary bacterial infections, such as pneumonia, and is often associated with other respiratory viruses and bacteria [1,4,5,6]. The severity of its clinical signs varies, with some animals, particularly young calves and the immunocompromised, experiencing more acute manifestations, while others may be non-clinical carriers [1]. BPI3V is one of the major contributors to the syndrome of bovine respiratory disease complex (BRDC) [7,8]. BRDC, often referred to as ‘shipping fever’ or ‘pneumonic pasteurellosis’, is a multifactorial syndrome resulting in considerable economic losses to the cattle industry, primarily due to reduced weight gain, decreased milk production, and increased veterinary costs [9,10]. Due to its potential to adversely affect cattle health, productivity, and general well-being, particularly in calves with poor passive transfer or decayed maternal antibodies, BPI3V has been a source of concern [1]. 

While many pathogens, including viruses and bacteria, have been implicated in BRDC, BPI3V has been noted for its ubiquitous presence across diverse geographic regions and cattle populations [11]. Due to its intricate interactions with other pathogens and various environmental and host factors, understanding the unique features of BPI3V infection is challenging [3,12,13,14,15,16,17]. However, the detection of BPI3V in BRDC cases [3,18] highlights its potential significance and role in the pathogenesis and progression of the syndrome.

BPI3V is primarily a respiratory pathogen in cattle [19,20,21] and is transmitted via aerosol within the population [22]. Based on genetic and phylogenetic analyses, BPI3V is classified into three genotypes (BPI3Va-c) [23,24,25]. Although it has been detected in healthy cattle and those with signs of BRDC [17], the specific role the virus plays in BRDC pathogenesis remains unclear, raising questions about its virulence and potential synergistic interactions with other pathogens. The fact that BPI3V has been identified in apparently healthy cattle suggests that its mere presence is not synonymous with clinical syndromes. Hence, due to its lower detection rate relative to other BRDC viruses [26,27] and the absence of experimental evidence supporting its role in BRDC pathogenesis, BPI3V is often considered less significant.

Despite the acknowledged prevalence of BPI3V and its connection with BRDC [3,18], there is a gap in dedicated research efforts focusing solely on BPI3V. A considerable amount of research on BPI3V is either incorporated within the broader context of BRDC or pivots towards detecting other prevalent BRDC pathogens. Moreover, the current research on BPI3V is fragmented, focusing primarily on regional prevalence and specific conditions, and lacks a global perspective. This highlights the need for a comprehensive synthesis of data on the prevalence of BPI3V across various geographical areas and cattle production systems.

A comprehensive understanding of the prevalence and distribution of BRDC pathogens, particularly BPI3V, is pivotal for devising effective management strategies for this syndrome. Indeed, detection methods that target antibodies, antigens, nucleic acids, and virus isolation (VI) offer varied insights into the prevalence rates [28,29]. The diagnostic landscape for this virus is diverse, with methods ranging from antibody-based assays that indicate past or current infections, to antigen detection techniques and more advanced nucleic acid-based approaches, like PCR and next-generation sequencing [26,27,28,30,31]. These techniques offer insights into the genetic composition and virulence of the pathogen, but each can present a different prevalence, depending on the sensitivity, specificity, or stage of infection they best detect. Therefore, to determine the prevalence of BPI3V and its role within the BRDC spectrum, the disparities in prevalence rates derived from different methodologies highlight the need for a comprehensive review. This study aimed to conduct a systematic review and meta-analysis of BPI3V prevalence to assess the variability in detection methods across different geographical regions and cattle populations based on the synthesis of the existing data, thereby exploring the complexities and associated risk factors of these methodologies.

## 2. Materials and Methods

### 2.1. Literature Search Strategy

The research questions, search and screening protocols, and result reporting followed the Preferred Reporting Items for Systematic Reviews and Meta-Analyses (PRISMA) guidelines [32]. Detailed information on the protocol can be found on OSF (registration https://doi.org/10.17605/OSF.IO/32Y5M). A uniform search strategy was developed, tested, and used to identify the relevant literature across three databases: Web of Science, PubMed, and Scopus. For a comprehensive search of relevant articles, the following search term was designed: (((((“Bovine parainfluenza-3 virus”) OR “BPI-3V”) OR “BPI3V”) OR “BPIV3”) OR “Bovine parainfluenza type 3 virus”). This term was then adapted to suit the advanced search criteria of each database. Specifically, PubMed employed the term as is; Web of Science used the tailored query TS = (((((“Bovine parainfluenza-3 virus”) OR “BPI-3V”) OR “BPI3V”) OR “BPIV3”) OR “Bovine parainfluenza type 3 virus”); and Scopus was queried with TITLE-ABS-KEY (((((“Bovine parainfluenza-3 virus”) OR “BPI-3V”) OR “BPI3V”) OR “BPIV3”) OR “Bovine parainfluenza type 3 virus”). This search protocol enabled the systematic download of relevant articles published until 1 June 2023 at 1:00 pm (Australian Central Daylight Time).

The retrieved articles were screened based on the inclusion and exclusion criteria described below. For inclusion, the focus was on articles with designs that were either cross-sectional, longitudinal, or case reports. The target population was cattle, covering any region, breed, age, or production system. The primary outcome measures centred on the prevalence or detection rate of BPI3V. Articles published primarily in English were considered; however, other languages were included if translations were available. Only articles that analysed more than 30 cattle and with at least two herds were considered. The exclusion criteria ruled out reviews, editorials, commentaries, and opinion articles. Articles targeting non-cattle species or those that did not provide prevalence or detection rates of BPI3V without lab confirmation were excluded. Furthermore, articles with less than 30 samples, lacking details about the origin and type of data used for analysis, or presenting duplicate or overlapping data were excluded. In addition to using PubMed, Web of Science, and Scopus, a manual search was carried out on Google Scholar to capture any missing articles.

### 2.2. Data Extraction and Quality Assessment

Using the Covidence platform (https://app.covidence.org/, accessed on 30 August 2023), the initial two authors methodically retrieved, reviewed, extracted, and ensured the quality of all articles. Discrepancies were addressed and resolved by consulting a third senior author. Data were systematically extracted from all included articles, targeting specific variables such as the author list, publication year, country of origin, farming system type (such as beef, dairy, or others), study design, sample type, and BPI3V detection method. The total number of cattle or herds tested, the total number of BPI3V-positive cattle or herds, and the age distribution of the cattle under investigation were also extracted. The data finalized through consensus were then incorporated into the analysis. For articles involving cohort or observational studies, data taken on day 0 were included in the current analysis. Articles reporting different BPI3V detection methods were considered as distinct entries in the meta-analysis, provided all samples originated from the same initial study population. Entries lacking clear descriptions of their sampling strategies and population types were not included in the final analysis. The Joanna Briggs Institute Qualitative Assessment and Review Instrument (JBI-QARI) tool was employed to evaluate the quality of the selected articles (available at https://jbi.global/critical-appraisal-tools, accessed on 30 August 2023).

### 2.3. Qualitative Data Selection and Analysis

To analyse qualitative data, all relevant articles were imported into the NVivo software [33] for thematic-based data extraction and evaluation. From the articles that reported risk factors and associated variables for BPI3V detection, NVivo was employed to systematically identify, code, and thematically categorize these factors. The organized data were exported after coding for further analysis.

### 2.4. Statistical and Meta-Analyses

Detection methods were categorized into three categories based on the identified analyte. The methods for detecting antibodies encompassed agar gel immunodiffusion (AGID), competitive ELISA, indirect ELISA, and neutralization tests. Under the antigen detection category, antigen capture ELISA, direct ELISA, the direct fluorescence antibody test (DFAT), immunohistochemistry, and sandwich ELISA were included. Nucleic acid detection incorporated techniques such as nested RT-PCR, real-time RT-PCR, and reverse transcriptase polymerase chain reaction (RT-PCR). The categorization of competitive ELISA—as either an antibody or antigen detection method—was based on the specific analyte identified in each article.

The study analysed sample types such as nasal swabs, bronchoalveolar lavage, lung tissue, and blood. Samples described in the literature as deep nasopharyngeal, nasopharyngeal, or nasal swabs were grouped into a single category labelled ‘nasal swab samples’. Similarly, those documented as lung lavage, tracheobronchial lavage, or bronchoalveolar lavage were classified under the ‘bronchoalveolar lavage samples’ category. For the meta-analysis, the study population was categorized into three groups: “Randomly Selected Samples”, comprising animals with no known history or signs of respiratory symptoms; “Observational (Intervention and Control)”, consisting of animals involved in longitudinal studies, including both observational and case–control studies; and “BRDC Cases”, which included samples collected from animals with respiratory symptoms, cattle populations identified as having BRDC in the primary study, and BRDC outbreaks.

The ‘meta’ and ‘metafor’ packages in R [34] were used for the meta-analysis. The meta-analysis employed the inverse variance method with the DerSimonian–Laird estimator for tau^2^. The prediction intervals were based on the t-distribution, and a Freeman–Tukey double arcsine transformation was used for the analysis [35]. Prevalence was estimated using a random-effects model and is presented as proportions accompanied by their 95% confidence intervals (CIs). The potential existence of publication bias was evaluated with Peters’ [36] and Egger’s [37] regression tests, and *p* < 0.05 was taken as significant.

The primary effect measure was identified as a pooled proportion, calculated by taking the ratio of cattle testing positive for BPI3V to the total cattle tested, represented with a 95% confidence interval. Given the anticipated heterogeneity across articles, a random-effects model was employed in the aggregated analysis. The degree of heterogeneity was assessed using the I^2^ statistic [38], complemented by the Q test [39].

Potential sources of heterogeneity were investigated through subgroup analysis, focusing on the detection method (antibody, antigen, or nucleic acid), animal category (beef cattle, dairy cattle, or local breeds), and health status (BRDC, subclinical, etc.). These subgroups were determined using predetermined variables like the type of intervention and the design of the study.

Effect size distribution was visually assessed using a Graphic Display of Heterogeneity (GOSH) [40]. As epidemiologic data frequently have a non-normal distribution, the DBSCAN algorithm was employed in outlier detection [41]. Outliers identified post-GOSH analysis, through both DBSCAN and Influence analysis, were consistently removed from the dataset. Following the outlier removal, a sensitivity analysis was conducted to evaluate the stability of the findings. After outlier exclusion, the meta-analysis was reiterated to confirm the consistency of the results.

A mixed-effects meta-regression model using the restricted maximum likelihood (REML) estimation approach was employed to identify the sources of heterogeneity across the included articles. This model probed potential variability among studies by incorporating several moderators: study population, country of origin, sample type, detection method, study design, farming system type, and cattle age group. Once the model was established, the coefficients and highlighted predictors that registered a *p*-value below 0.05 were extracted.

## 3. Results

### 3.1. Study Selection and Characteristics

**Figure 1:**
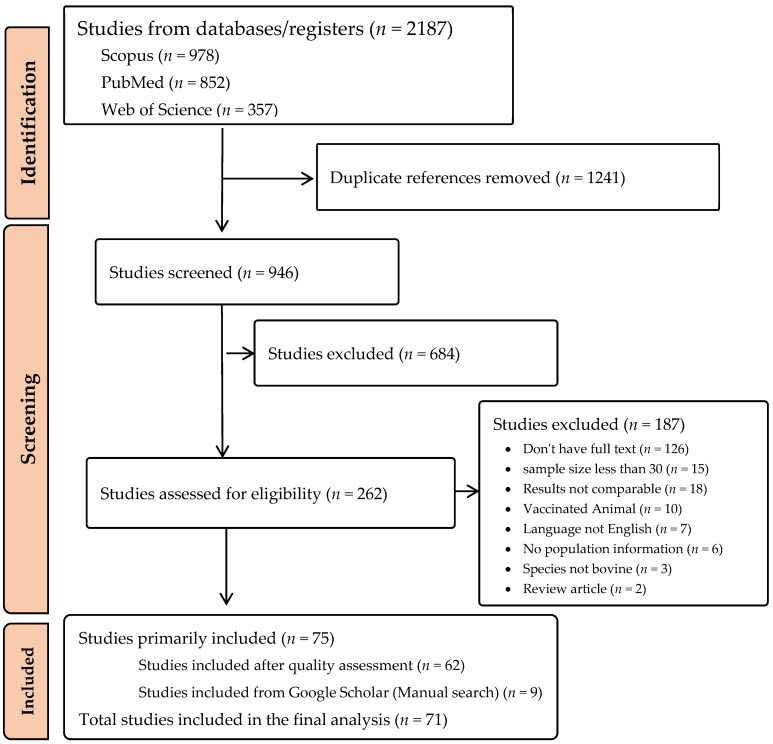
Steps followed for data collection and screening.

**Figure 2:**
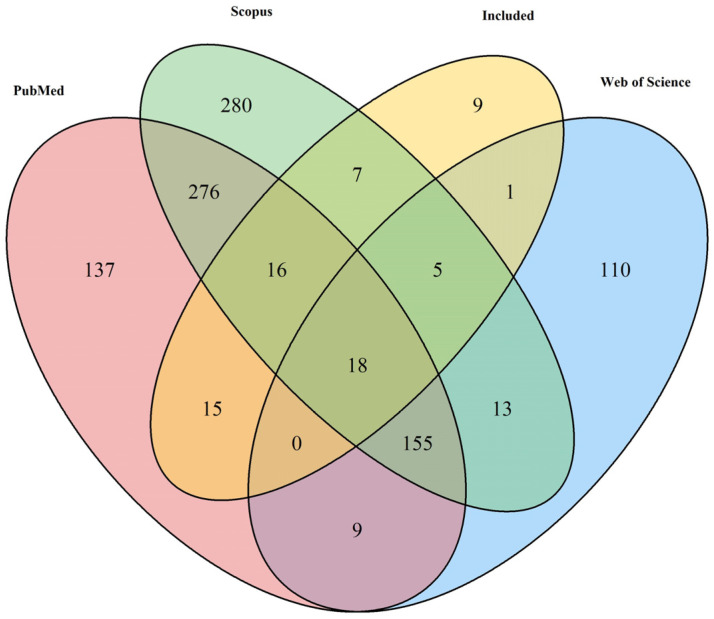
A Venn diagram illustrating the intersection of articles retrieved from PubMed, Web of Science (WoS), Scopus, and Google Scholar, highlighting those chosen for meta-analysis (labelled as Included).

In this study, a total of 2187 articles were initially retrieved, 946 were screened after duplications were removed, and 62 were included in the final analysis (Figure 1). An exception to this data extraction process was a group of 9 articles that were manually retrieved from Google Scholar, bringing the number of articles included in this study to 71 (Figure 2). Of the 71 articles included, 9 were also selected for qualitative analysis. From the 71 articles analysed, only 33 specifically mentioned “Parainfluenza” in their title. The remaining articles mainly focused on BRDC or its associated pathogens, incorporating BPI3V into their findings but not being their main research focus area. The meta-analysis included articles from 32 countries. While 71 articles were included in this study, some of these articles used multiple detection methods for BPI3V. Hence, in terms of different detection methods, these articles contribute multiple times, resulting in 75 distinct data entries derived from the 71 articles. The included articles were further categorized for subgroup analysis (Figures S1–S5).

### 3.2. Prevalence of Parainfluenza-3 Virus in Cattle

**Figure 3:**
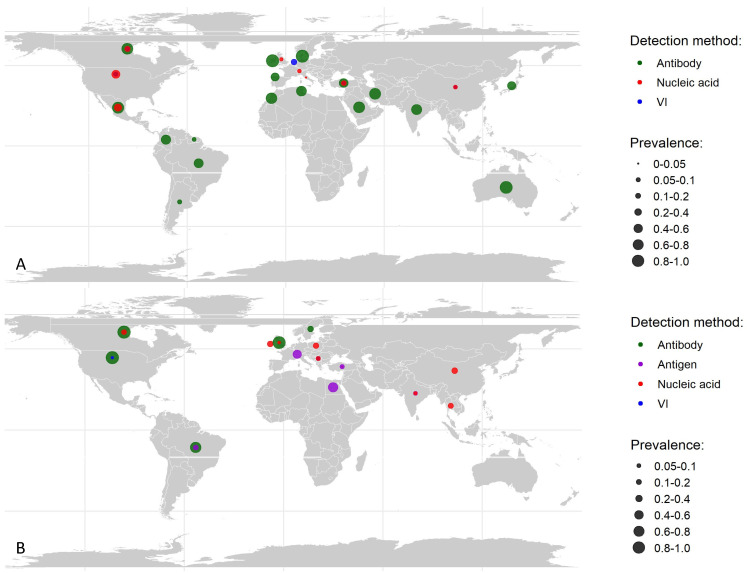
Global distribution of bovine parainfluenza-3 virus in different cattle populations. (**A**) Prevalence in the general cattle population, where detection was carried out through antibody testing, nucleic acid assays, and virus isolation (VI) methods. (**B**) Prevalence in cattle with bovine respiratory disease complex, using detection methods such as antibody, antigen and nucleic acid testing, and VI.

With varying prevalences, BPI3V was found distributed globally (Figure 3). Before conducting the meta-analysis, the dataset was systematically divided into two categories based on the study population specified in the primary articles: one focusing on the prevalence of BPI3V in the general cattle population and the other on cattle diagnosed with BRDC (Figure 3).

In the pooled prevalence of BPI3V in the general cattle population, 48 articles involving 27,149 observations and 16,461 events were analysed. In the general cattle population, before excluding outliers, a pooled prevalence of 0.36 (95% CI: 0.25 to 0.48) was observed. For the pooled prevalence of BPI3V in cattle with BRDC, 27 articles, consisting of 6398 observations and 1422 events, were analysed. The pooled prevalence of BPI3V in cattle with BRDC was 0.20 (95% CI: 0.10 to 0.32).

In both the general cattle population and cattle with BRDC, subgroup analysis showed significant differences (*p* < 0.05) in the prevalence of BPI3V among the detection methods used. However, no significant difference in the prevalence of BPI3V was observed between the general cattle population and cattle with BRDC. In each meta-analysis described here, consisting of at least 10 studies, publication bias was assessed using Egger’s and Peter’s tests, and no publication bias was detected.

#### 3.2.1. Prevalence of Bovine Parainfluenza-3 Virus Detected by Antibody-Based Detection Methods

**Figure 4:**
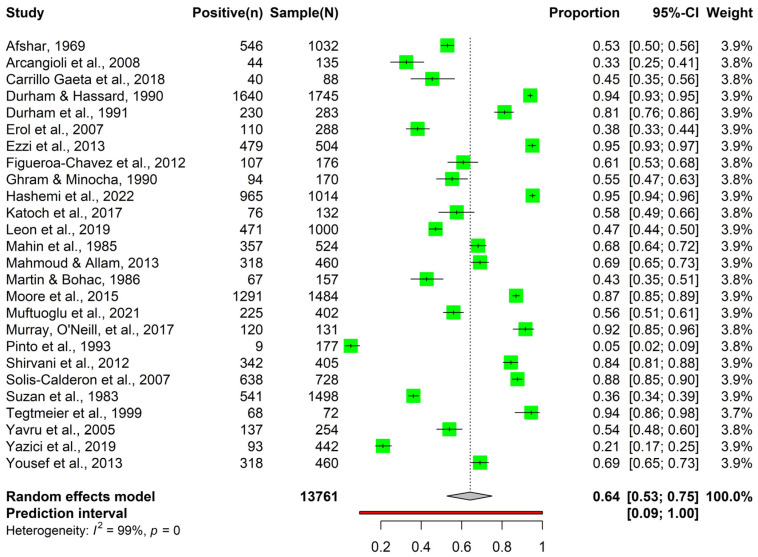
Meta-analysis output on the prevalence of bovine parainfluenza-3 virus observed using antibody detection methods (such as agar gel immunodiffusion, competitive ELISA, indirect ELISA, and neutralization tests) [12,17,43,46,47,48,49,50,51,52,53,54,55,56,57,58,59,60,61,62,63,64,65,66,67,68].

Using the antibody detection method, the meta-analysis was carried out using 30 articles containing 22,297 observations and 16,082 events. The individual article proportions ranged from 0.04 to 0.95. Specifically, the lowest prevalence was reported by Virakul et al. (1985) [42] at 0.04, while the highest prevalence was observed in the article by Hashemi et al. (2022) [43] at 0.95 (Figure 4, Figure S1 and Table 1).

**Table 1:** Prevalence of bovine parainfluenza-3 virus using antibody detection method before and after outlier removal.

Criteria	Before Outlier Removal	After Outlier Removal
Number of articles	30	26 *
Number of observations	22,297	13,761
Number of events	16,082	9326
Pooled prevalence	0.60 (95% CI: 0.47 to 0.71)	0.64 (95% CI: 0.53 to 0.75)
Prediction interval	0.04 to 1.00	0.09 to 1.00
Heterogeneity (I^2^)	99.7% (tau^2^ = 0.11, H = 17.04)	99.4% (tau^2^ = 0.09, H = 13.25)

* outliers: [15,42,44,45].

After outlier detection and removal, a total of 26 articles were included, consisting of 13,761 observations and 9326 events. The proportions of BPI3V prevalence varied significantly across the articles, with individual article proportions ranging from as low as 0.05 in Pinto et al., 1993 [46] to as high as 0.95 in Hashemi et al., 2022 [43]. The pooled prevalence estimate was 0.64 (95% CI: 0.53 to 0.75) (Figure 4 and Table 1). In the subgroup analysis using the antibody detection method, significant differences (*p* < 0.05) were observed based on age groups and farm types but not within study designs or population characteristics (Figure S1A–C).

#### 3.2.2. Prevalence of Bovine Parainfluenza-3 Virus Detected by Nucleic Acid-Based Detection Methods

Initially, the meta-analysis on the prevalence of BPI3V using nucleic acid detection methods consisted of 12 articles involving 3405 observations and 281 events. The individual article proportions ranged from 0.00 (Padalino et al., 2021 [69]) to 0.34 (Sarchet et al., 2022 [70]) (Table 2).

**Figure 5:**
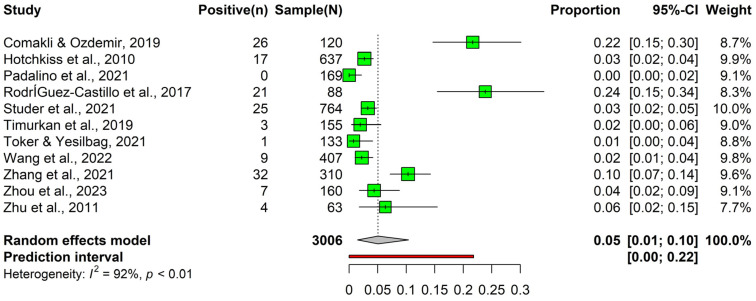
Forest plot of the prevalence of bovine parainfluenza-3 virus observed using nucleic acid detection methods (such as nested RT-PCR, real-time RT-PCR, and RT-PCR) [5,9,28,69,71,72,73,74,75,76,77].

A pooled prevalence of 0.07 (95% CI: 0.02 to 0.14) and a prediction interval from 0.00 to 0.39 were observed. Following the outlier detection and removal, the meta-analysis included 11 articles with 3006 observations and 145 events. Post-outlier removal, the pooled prevalence reduced to 0.05 (95% CI: 0.01 to 0.10) and showed a prediction interval of 0.00 to 0.22 (Table 2). While heterogeneity remained significant, it was reduced compared to that in the initial analysis, with an I^2^ of 92.2%, a tau^2^ of 0.01, and a tau of 0.11. The test for heterogeneity continued to be significant (Q = 127.97, df = 10, *p* < 0.05) (Figure 5, Figure S2 and Table 2). Subgroup analysis showed a statistically significant difference (*p* < 0.05) between different age groups. However, the analysis did not show any significant differences when comparing various farming systems. Additionally, the number of articles included for subgroup analysis based on study design was insufficient to make a difference (Figure S2).

#### 3.2.3. Prevalence of Bovine Parainfluenza-3 Virus Detected by Virus Isolation

**Figure 6:**
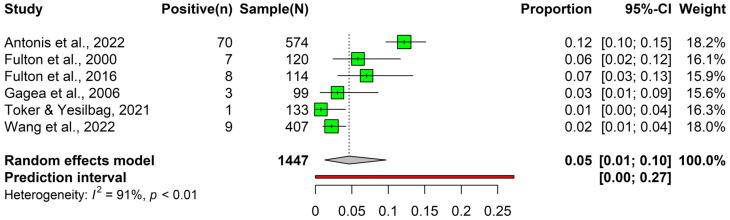
Forest plot of the prevalence of bovine parainfluenza-3 virus observed using virus isolation [27,75,76,78,79,80].

In the general cattle population, a meta-analysis was carried out on six articles consisting of 1447 observations and 98 events to evaluate the prevalence of BPI3V using VI methods. The article-specific prevalence of BPI3V varied considerably, with the lowest being 0.01 (Toker & Yesilbag, 2021 [75]) and the highest being 0.12 (Antonis et al., 2022 [78]). The pooled prevalence estimate was calculated as 0.05 (95% CI: 0.01 to 0.10) (Figure 6). Due to the limited number of articles available, subgroup analysis for BPI3V detection through virus isolation methods could not be conducted.

### 3.3. Bovine Parainfluenza-3 Virus Prevalence among Bovine Respiratory Disease Complex (BRDC) Cases

The meta-analysis focused on the detection rate of BPI3V in cattle with BRDC and examined the variation in detection methods across 27 articles, incorporating 6398 observations and 1422 events. The pooled prevalence was 0.20 (95% CI: 0.10 to 0.32). Significant heterogeneity was observed in the overall analysis, indicated by an I^2^ value of 99.3%, a tau^2^ of 0.15, a tau of 0.39, and an H statistic of 11.79 (Q = 3611.44, df = 26, *p* < 0.05). The test for detection method subgroup differences was significant (Q = 20.33, df = 3, *p* < 0.05). A separate meta-analysis was carried out for each method employed to detect BPI3V.

The prevalence of BPI3V in cattle with BRDC was assessed through VI methods, involving only two articles with 218 observations and 10 events [81,82]. The analysis observed a pooled BPI3V prevalence of 0.04 (95% CI: 0.00 to 0.64).

#### 3.3.1. Prevalence of Bovine Parainfluenza-3 Virus Using Antibody Detection Methods in Cattle with Bovine Respiratory Disease Complex

**Figure 7:**
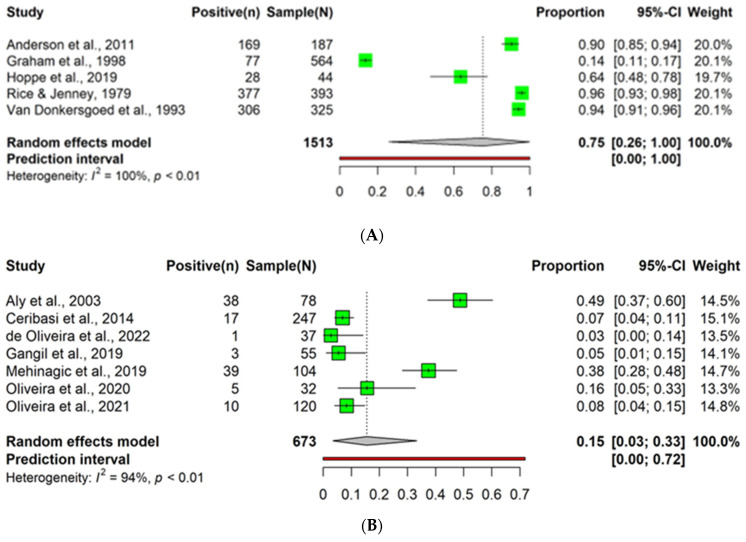

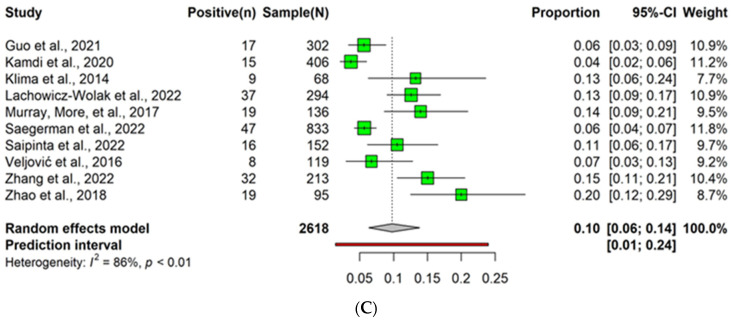
Forest plots of the prevalence of bovine parainfluenza-3 virus in cattle affected by bovine respiratory disease complex, as observed by the following methods: (**A**) antibody (such as agar gel immunodiffusion, competitive ELISA, indirect ELISA, and neutralization tests) [83,84,85,86,87], (**B**) antigen (such as antigen capture ELISA, direct ELISA, the direct fluorescence antibody test (DFAT), immunohistochemistry, and sandwich ELISA) [6,20,88,89,90,91,92], and (**C**) nucleic acid methods (such as nested RT-PCR, real-time RT-PCR, and RT-PCR) [16,82,93,94,95,96,97,98,99,100].

A meta-analysis was conducted on BPI3V prevalence detected using antibody methods in cattle with BRDC. The analysis exclusively consisted of studies on calves, with no adult cattle included. Additionally, within the farming system context, the analysis included merely two articles that specifically focused on dairy cattle, and no studies on beef cattle were included. Five articles (two diagnostic test accuracy tests and three BRDC cases), consisting of 1513 observations and 957 events, were included in the analysis. The individual article proportions for BPI3V prevalence ranged notably from a low of 0.14 (Graham et al., 1998 [83]) to a high of 0.96 (Rice & Jenney, 1979 [84]). The pooled prevalence was 0.75 (95% CI: 0.26 to 1.00). The prediction interval was extremely wide, ranging from 0.00 to 1.00 (Figure 7A and Figure S3).

#### 3.3.2. Prevalence of Bovine Parainfluenza-3 Virus Using Antigen Detection Methods in Bovine Respiratory Disease Complex Cases

In this meta-analysis, the prevalence of BPI3V in cattle was assessed using antigen-based detection methods. The analysis included seven articles, with 673 observations and 113 events. The pooled prevalence of BPI3V was 0.15 (95% CI: 0.03 to 0.33). The analysis observed a high level of heterogeneity among the included articles, with an I^2^ of 94.3%, a tau^2^ of 0.05, a tau of 0.22, and a significant test for heterogeneity (*p* < 0.05). The individual articles observed varying prevalence rates, with the highest prevalence reported by Aly et al., 2003 [88] at 0.49 and the lowest by de Oliveira et al., 2022 [89] at 0.03 (Figure 7B).

The subgroup analysis based on farming systems showed that the dairy farming system observed a prevalence of 0.32, while the mixed or unknown farming system observed a prevalence of 0.11. The test for differences between these farming systems was not significant (*p* > 0.05) (Figure S4).

#### 3.3.3. Prevalence of Bovine Parainfluenza-3 Virus Using Nucleic Acid Detection Methods in Bovine Respiratory Disease Complex Cases

The meta-analysis examined the detection rate of BPI3V in cattle using nucleic acid methods, both before and after outlier detection and removal. Initially, the analysis included 13 articles with 3994 observations and 342 events. The individual article proportions ranged from 0.01 (Küçük & Yildirim, 2022 [101]) to 0.20 (Zhao et al., 2018 [100]). A pooled prevalence of 0.09 (95% CI: 0.05 to 0.13) with an I^2^ of 92.7%, a tau^2^ of 0.01, and a tau of 0.10 was observed (Q = 163.68, df = 12, *p* < 0.05) (Figure 7C and Table 3). After removing outliers, the analysis included 10 articles comprising 2618 observations and 219 events. Post-outlier removal, the pooled prevalence was slightly higher at 0.10 (95% CI: 0.06 to 0.14) (Table 3). There was a reduction in heterogeneity compared to that in the initial analysis, with an I^2^ of 85.6%, a tau^2^ of 0.006, and a tau of 0.08 (Q = 62.63, df = 9, *p* < 0.05) (Table 3).

The subgroup analysis indicated no significant differences in BPI3V prevalence across age groups or farming systems. In adult cattle (two articles), the pooled prevalence was 0.15 (95% CI: 0.00 to 0.76), and in calves (three articles), it was 0.09 (95% CI: 0.00 to 0.27). For farming systems, the beef subgroup (two articles) observed a prevalence of 0.08 (95% CI: 0.00 to 0.85), with notable heterogeneity (I^2^ = 76.3%). The dairy subgroup, with six articles, had a slightly higher prevalence of 0.10 (95% CI: 0.05 to 0.17) and also high heterogeneity (I^2^ = 90.4%). The mixed/unknown farming systems, covering two articles, observed a prevalence of 0.10 (95% CI: 0.00 to 0.78), with significant heterogeneity (I^2^ = 71.6%). No statistical significance was found between these farming subgroups (*p* > 0.05) (Figure S5A,B).

Different study designs—such as case series and diagnostic test accuracy—also revealed varying prevalence. The case series subgroup (eight articles) observed a prevalence of 0.08 (95% CI: 0.05 to 0.12) with substantial heterogeneity (I^2^ = 80.8%), whereas the diagnostic test accuracy subgroup (two articles) reported a higher prevalence of 0.17 (95% CI: 0.00 to 0.54) with lower heterogeneity (I^2^ = 16.1%). A significant difference was noted between these subgroups (*p* < 0.05), suggesting that article design considerably impacts BPI3V prevalence estimates (Figure S5C).

### 3.4. Factors Influencing Prevalence of Parainfluenza Type 3 Virus

The mixed-effects model analysis, utilizing a dataset from 74 entries and employing the REML method for tau^2^ estimation, offered a detailed assessment of factors influencing bovine parainfluenza-3 virus (BPI3V) prevalence. The model revealed a moderate level of residual heterogeneity (tau^2^ = 0.01, SE = 0.01) with the square root of tau^2^ at 0.10 and a high I^2^ value of 93.67%, indicating that a significant portion of the variability in the effect size was due to heterogeneity. The H^2^ value stood at 15.80, with the model accounting for 91.62% of the heterogeneity. The presence of significant residual heterogeneity was confirmed (QE (df = 1) = 15.80, *p* < 0.05).

In the test of moderators, significant influences were observed across coefficients 2 to 73 (QM (df = 72) = 837.57, *p* < 0.05). Specifically, country-specific effects emerged as a significant factor, with countries including Australia, Brazil, Canada, China, and others showing positive associations with BPI3V prevalence, ranging from 1.47 in the UK to 6.21 in Morocco. The prevalence was also significantly influenced by detection methods and age groups, with the mixed or unknown age group showing a negative association (estimate = −1.10). Moreover, the year of study proved to be a crucial predictor, with specific years, including 1985 and 2010, negatively impacting BPI3V prevalence. The model also highlighted interactions between the study population and country; for example, the ‘Apparently healthy’ population in China correlated with a decreased prevalence of BPI3V (estimate = −0.58).

The present study identified risk factors that could potentially impact the prevalence of BPI3V. The identified risk factors included the age of the cattle, coinfection, farm type, sex, respiratory signs, and body weight (Table 4).

## 4. Discussion

This systematic review and meta-analysis, to the best of our knowledge, provided the first quantitative estimation of global BPI3V prevalence. The study provided a comprehensive analysis of the prevalence of BPI3V across different geographic regions and study populations, employing diverse detection methods.

The meta-analysis revealed significant variations in BPI3V prevalence depending on the detection method used. In the general cattle population and cattle with BRDC, the highest BPI3V prevalence was observed using the antibody detection method, with a proportion of 0.64 and 0.74, respectively. The higher prevalence of BPI3V in BRDC cattle (0.74 compared to 0.64 in the general cattle population) might reflect an earlier and potentially more widespread exposure to the virus in this group. BRDC-affected cattle could have been exposed to the virus long enough for antibodies to develop, perhaps due to factors such as weakened immune defences or stressful environmental conditions, which are conducive to viral transmission and infection [1,104]. For the antigen detection method, a prevalence of 0.15 was observed exclusively in cattle with BRDC. Using nucleic acid detection, a prevalence of 0.05 and 0.10 was observed in the general and BRDC cattle populations, respectively, indicating a significant population-based difference in the detection rate of BPI3V. The lower prevalence rates observed through nucleic acid detection as compared to antibody prevalence (discussed in a previous query) indicate that while a significant portion of the cattle population has been exposed to BPI3V in the past (as shown by antibodies), a smaller fraction currently harbours the virus. VI methods indicated a prevalence of 0.05 in the general cattle population and of 0.04 in cattle with BRDC, demonstrating a comparable rate of active BPI3V infection across both groups. These findings highlight the differences in the detection ability of different methods in identifying BPI3V. Moreover, the differences in prevalence can be attributed to the sensitivities and specificities inherent to each detection method [31,105], which could also be affected by genotype and/or antigenic differences in the virus [24,25].

Compared to nucleic acid detection, virus isolation, and antigen detection methods that detect active infections, antibody tests typically measure past exposure and do not always indicate an active infection [1,106], resulting in higher apparent prevalence rates. However, given the endemic status of BPI3V in many herds or countries, the diagnostic significance of a single serum sample is optimally demonstrated through the levels of IgM and IgA antibodies, indicative of recent or reinfection [107]. As viral shedding occurs for a limited duration [108], before deciding which detection method to use, it is important to consider the epidemiology of the virus.

Despite these disparities, this meta-analysis did not show differences in the prevalence of BPI3V amongst the different study population subgroups, suggesting the prevalence of BPI3V was relatively consistent, regardless of the health status of the study population (i.e., apparently healthy, BRDC-affected, etc.), study design, production type, and sample type. Moreover, adult cattle observed a higher prevalence of BPI3V, similar to previous reports [53,56,63,102], potentially hinting at a cumulative exposure over time or increased susceptibility with age.

In this study, given the unexplained high heterogeneity in the meta-analysis results, a meta-regression analysis was undertaken, revealing negligible unaccounted heterogeneity. The results of the meta-regression analysis showed that various factors, including study populations, geographic regions, sample types, detection methods, years, and geographic locations, played a substantial role in the prevalence of BPI3V. This finding was similar to those in previous reports on the detection rates of BPI3V by geographic location [23,24,25,30], sample type [107,109], and detection method [31].

However, the current study observed differences between the meta-regression and subgroup meta-analysis findings. While the meta-regression model highlighted multiple variables as significant influencers of BPI3V prevalence, such as geographic region and study population, the subgroup meta-analysis using a random-effects model showed statistical significance solely for detection methods and age variables. Several factors could contribute to this incongruence. Methodological differences between the two analytical approaches, varying statistical power, and the sensitivity to study heterogeneity could all play roles. Specifically, meta-regression offers a broader evaluation of potential moderators by handling continuous and categorical variables [110].

In contrast, subgroup meta-analysis isolates the impact of specific categorical variables [111]. Additionally, the increased sensitivity of meta-regression to study heterogeneity may enable it to capture subtle, interactive effects among variables that could be overlooked in a more constrained subgroup analysis [110]. The observed disparities underscore the complexity of factors affecting BPI3V prevalence and highlight the need for further research to reconcile these divergent findings and deepen our understanding of BPI3V epidemiology.

BPI3V can be detected in either healthy cattle or those with signs of BRDC, mostly accompanying other pathogens [1,17,24,81], further complicating the detection rate and epidemiology of BPI3V. Subclinical infection with BPI3V plays a role in sustaining the presence of the virus in cattle populations [1]. Moreover, articles on BPI3V frequently come from larger BRDC studies or those focusing primarily on other pathogens like BVDV, BHV1, or *Mycoplasma bovis* [6,26]. The detection of BPI3V alongside several pathogens within or without BRDC and their complex interactions make it difficult to identify the exact role of BPI3V in BRDC [15,16,59,112]. A complete understanding of BPI3V and its risk factors is yet lacking, as evidenced by the layered complexity of the pathogens linked to BRDC. The heterogeneity observed across the included articles was likely caused by the complexities of BRDC pathogens and study characteristics.

Several risk factors for BPI3V were identified in this study. Interestingly, adult cattle were associated with a higher seropositive rate [53,56,63], a finding consistent with other respiratory diseases where older animals have prolonged exposure [105]. Within a similar age group, calves with lower body weights were associated with a higher prevalence of BPI3V [103], a phenomenon that might be attributable to the potential immunosuppressive effects of the virus on calves with low body condition scores [1]. Hence, seroconversion rates to BPI3V could be used as a predictor of reduced weight gain [113]. Females had a higher prevalence of BPI3V than males [56], suggesting physiological or immunity-related factors. Moreover, an association between the coinfection of BPI3V and BRSV was observed [55,58,68,112], suggesting potential synergistic effects or shared transmission routes between these viruses. BPI3V infects in synergy with other BRDC pathogens like *M. bovis* [5,6]. Larger farm sizes and having more older cattle on a farm were also linked to higher prevalence rates [63]. This might reflect increased exposure opportunities in larger populations or less stringent biosecurity measures in such setups. Unsurprisingly, cattle with respiratory signs were observed to have a higher prevalence of BPI3V, potentially emphasizing the clinical relevance of this virus [53]. In another study, cattle with prior exposure were found to have a reduced risk of BRDC [15]. However, reports on the association between BPI3V detection and BRDC are inconsistent [12,13,14,15,16] and, hence, require further research to understand the role of BPI3V in the pathogenesis of BRDC, particularly considering the potential confounding effects of factors like age, farm type, sex, and body weight on BPI3V prevalence.

This review did not come without some limitations. While no publication bias was detected using the antibody or antigen detection methods, there was evidence of such bias in studies employing nucleic acid detection methods, indicating that there may be unpublished studies with contradictory or insignificant results, which could skew our understanding of the prevalence of BPI3V.

## 5. Conclusions

This systematic review and meta-analysis indicated the widespread prevalence of BPI3V in cattle populations globally. The differences in prevalence, based on detection methods and other variables, call for a cautious interpretation of detection results. Given the complex epidemiology of BPI3V, particularly its role in BRDC and frequent coinfection with other pathogens, employing antibody and nucleic acid-based tests in tandem would provide the most comprehensive understanding of BPI3V prevalence and its role in cattle health. Furthermore, understanding the risk factors associated with BPI3V can assist in developing and implementing targeted interventions, leading to improved cattle health and farm productivity. Future research should also explore the role of BPI3V in BRDC and its associated risk factors, its interaction with other pathogens, and the potential effects of vaccination and other preventive strategies.

## Figures and Tables

**Table 2:** Prevalence of bovine parainfluenza-3 virus using nucleic acid detection methods before and after outlier removal.

Criteria	Before Outlier Removal	After Outlier Removal
Number of articles	12	11 *
Number of observations	3405	3006
Number of events	281	145
Pooled prevalence	0.07 (95% CI: 0.02 to 0.14)	0.05 (95% CI: 0.01 to 0.10)
Prediction interval	0.00 to 0.39	0.00 to 0.22
Heterogeneity (I^2^)	97.1% (tau^2^ = 0.03, H = 5.86)	92.2% (tau^2^ = 0.01, H = 3.58)
Test of heterogeneity (Q, *p*-value)	378.04, *p* < 0.05	127.97, *p* < 0.05

* outlier: [70].

**Table 3:** Prevalence of bovine parainfluenza-3 virus using nucleic acid detection methods in cattle with bovine respiratory disease complex before and after outlier removal.

Criteria	Before Outlier Removal	After Outlier Removal
Number of articles	13	10 *
Number of observations	3994	2618
Number of events	342	219
Pooled prevalence	0.09 (95% CI: 0.05 to 0.13)	0.10 (95% CI: 0.06 to 0.14)
Prediction interval	0.00 to 0.26	0.01 to 0.24
Heterogeneity (I^2^)	92.7% (tau^2^ = 0.01, H = 3.69)	85.6% (tau^2^ = 0.01, H = 2.64)

* outliers: [3,31,101].

**Table 4:** Risk factors associated with the prevalence of bovine parainfluenza-3 virus.

Risk Factor	Description	References
Age	Adult cattle were associated with a higher seropositive rate.	[53,56,63,102]
Coinfection	Bovine parainfluenza-3 virus detection was associated with bovine respiratory syncytial virus detection.	[55,58,68]
Farm type	Larger farm size was linked to a higher prevalence of the virus, as was	[63]
	farms with older animals and cattle not born on a farm.	[63]
Respiratory sign	Prevalence of bovine parainfluenza-3 virus was higher in cattle with respiratory signs.	[15,53]
Sex	Females showed higher prevalence compared to males.	[56]
Body weight	Within a similar age group, calves with lower body weights were associated with a higher prevalence of bovine parainfluenza-3 virus.	[103]

## Data Availability

Data can be available upon request to the authors.

## Supplementary Materials

The following supporting information can be downloaded at: https://www.mdpi.com/article/10.3390/ani14030494/s1, Figure S1: Subgroup analysis of bovine parainfluenza type 3 virus prevalence using antibody detection methods; Figure S2: Subgroup analysis of bovine parainfluenza type 3 virus prevalence using nucleic acid detection methods; Figure S3: Subgroup analysis of bovine parainfluenza type 3 virus prevalence in cattle with BRDC using antibody detection methods; Figure S4: Farming system-based subgroup analysis of bovine parainfluenza type 3 virus prevalence in cattle with BRDC using antigen detection methods; Figure S5: Subgroup analysis of bovine parainfluenza type 3 virus prevalence in cattle with BRDC using nucleic acid detection methods.
