# Supplementary material for: Bovine Parainfluenza-3 Virus Detection Methods and Prevalence in Cattle: A Systematic Review and Meta-Analysis
Source: Animals (Basel). 2024 Feb 2;14(3):494. doi: 10.3390/ani14030494 (PMC10854990; doi:10.3390/ani14030494)
Supplement: Supplementary file 1 [file animals-14-00494-s001.zip › animals-2798813-supplementary.pdf]

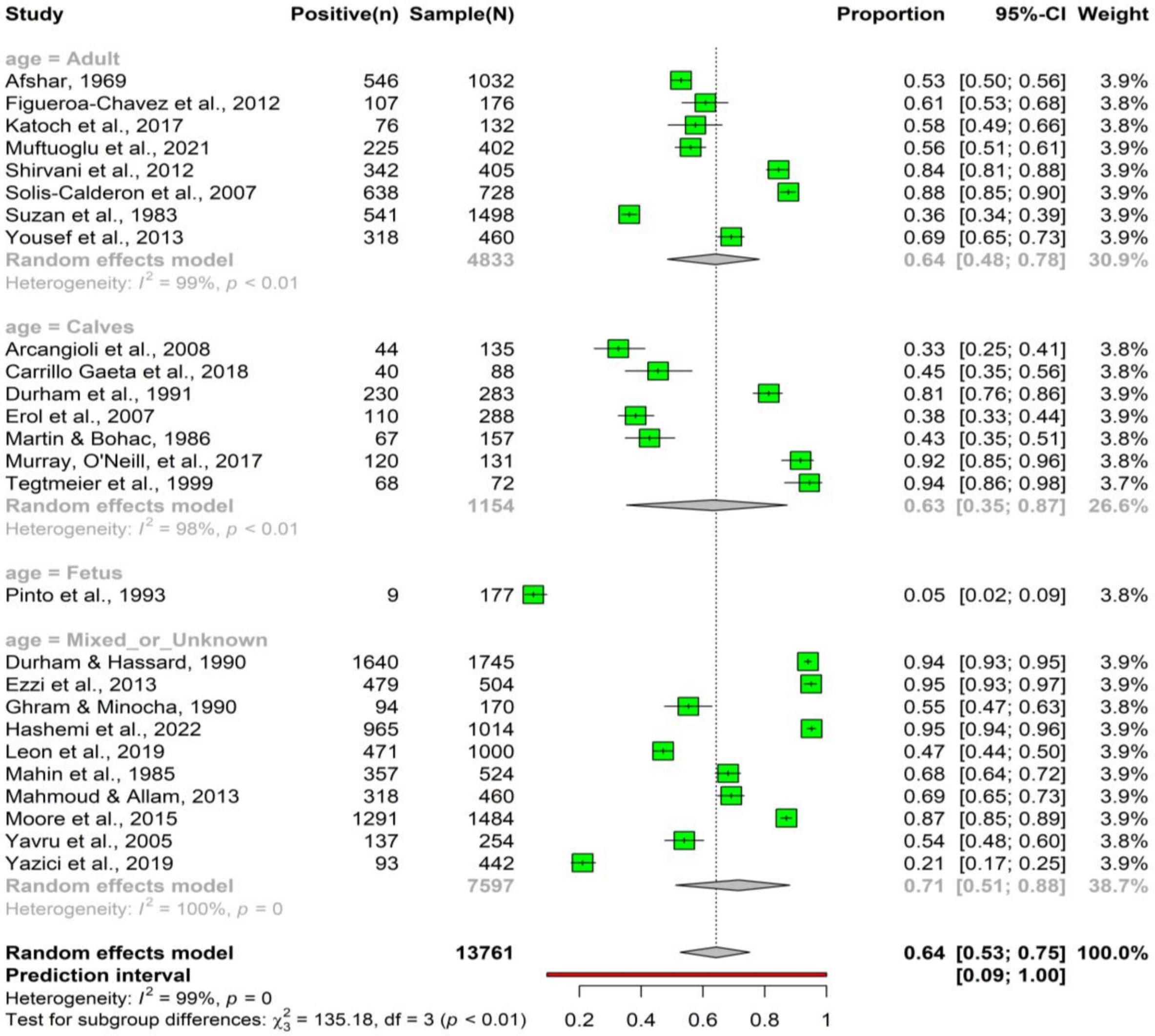
Study Positive(n) Sample(N) Proportion 95%-CI Weight
age = Adult
Afshar, 1969 546 1032 0.53 [0.50; 0.56] 3.9%
Figueroa-Chavez et al., 2012 107 176 0.61 [0.53; 0.68] 3.8%
Katoch et al., 2017 76 132 0.58 [0.49; 0.66] 3.8%
Muftuoglu et al., 2021 225 402 0.56 [0.51; 0.61] 3.9%
Shirvani et al., 2012 342 405 0.84 [0.81; 0.88] 3.9%
Solis-Calderon et al., 2007 638 728 0.88 [0.85; 0.90] 3.9%
Suzan et al., 1983 541 1498 0.36 [0.34; 0.39] 3.9%
Yousef et al., 2013 318 460 0.69 [0.65; 0.73] 3.9%
Random effects model 4833 0.64 [0.48; 0.78] 30.9%
Heterogeneity: I^2 = 99%, p < 0.01
age = Calves
Arcangioli et al., 2008 44 135 0.33 [0.25; 0.41] 3.8%
Carrillo Gaeta et al., 2018 40 88 0.45 [0.35; 0.56] 3.8%
Durham et al., 1991 230 283 0.81 [0.76; 0.86] 3.9%
Erol et al., 2007 110 288 0.38 [0.33; 0.44] 3.9%
Martin & Bohac, 1986 67 157 0.43 [0.35; 0.51] 3.8%
Murray, O'Neill, et al., 2017 120 131 0.92 [0.85; 0.96] 3.8%
Tegtmeier et al., 1999 68 72 0.94 [0.86; 0.98] 3.7%
Random effects model 1154 0.63 [0.35; 0.87] 26.6%
Heterogeneity: I^2 = 98%, p < 0.01
age = Fetus
Pinto et al., 1993 9 177 0.05 [0.02; 0.09] 3.8%
age = Mixed_or_Unknown
Durham & Hassard, 1990 1640 1745 0.94 [0.93; 0.95] 3.9%
Ezzi et al., 2013 479 504 0.95 [0.93; 0.97] 3.9%
Ghram & Minocha, 1990 94 170 0.55 [0.47; 0.63] 3.8%
Hashemi et al., 2022 965 1014 0.95 [0.94; 0.96] 3.9%
Leon et al., 2019 471 1000 0.47 [0.44; 0.50] 3.9%
Mahin et al., 1985 357 524 0.68 [0.64; 0.72] 3.9%
Mahmoud & Allam, 2013 318 460 0.69 [0.65; 0.73] 3.9%
Moore et al., 2015 1291 1484 0.87 [0.85; 0.89] 3.9%
Yavru et al., 2005 137 254 0.54 [0.48; 0.60] 3.8%
Yazici et al., 2019 93 442 0.21 [0.17; 0.25] 3.9%
Random effects model 7597 0.71 [0.51; 0.88] 38.7%
Heterogeneity: I^2 = 100%, p = 0
Random effects model 13761 0.64 [0.53; 0.75] 100.0%
Prediction interval [0.09; 1.00]
Heterogeneity: I^2 = 99%, p = 0
Test for subgroup differences: χ^2_3 = 135.18, df = 3 (p < 0.01)
0.2 0.4 0.6 0.8 1

A)

B)

Study	Positive(n)	Sample(N)	Proportion	95%-CI	Weight
**age = Beef**					
Arcangioli et al., 2008	44	135	0.33	[0.25; 0.41]	3.8%
Durham et al., 1991	230	283	0.81	[0.76; 0.86]	3.9%
Martin & Bohac, 1986	67	157	0.43	[0.35; 0.51]	3.8%
Moore et al., 2015	1291	1484	0.87	[0.85; 0.89]	3.9%
Solis-Calderon et al., 2007	638	728	0.88	[0.85; 0.90]	3.9%
**Random effects model**		**2787**	**0.69**	**[0.34; 0.95]**	**19.2%**
Heterogeneity: $I^2 = 99\%$, $p < 0.01$					
**age = Dairy**					
Carrillo Gaeta et al., 2018	40	88	0.45	[0.35; 0.56]	3.8%
Erol et al., 2007	110	288	0.38	[0.33; 0.44]	3.9%
Ezzi et al., 2013	479	504	0.95	[0.93; 0.97]	3.9%
Ghram & Minocha, 1990	94	170	0.55	[0.47; 0.63]	3.8%
Katoch et al., 2017	76	132	0.58	[0.49; 0.66]	3.8%
Shirvani et al., 2012	342	405	0.84	[0.81; 0.88]	3.9%
Yousef et al., 2013	318	460	0.69	[0.65; 0.73]	3.9%
**Random effects model**		**2047**	**0.66**	**[0.44; 0.85]**	**26.8%**
Heterogeneity: $I^2 = 99\%$, $p < 0.01$					
**age = Mixed_or_Unknown**					
Afshar, 1969	546	1032	0.53	[0.50; 0.56]	3.9%
Durham & Hassard, 1990	1640	1745	0.94	[0.93; 0.95]	3.9%
Figueroa-Chavez et al., 2012	107	176	0.61	[0.53; 0.68]	3.8%
Hashemi et al., 2022	965	1014	0.95	[0.94; 0.96]	3.9%
Leon et al., 2019	471	1000	0.47	[0.44; 0.50]	3.9%
Mahin et al., 1985	357	524	0.68	[0.64; 0.72]	3.9%
Mahmoud & Allam, 2013	318	460	0.69	[0.65; 0.73]	3.9%
Muftuoglu et al., 2021	225	402	0.56	[0.51; 0.61]	3.9%
Murray, O'Neill, et al., 2017	120	131	0.92	[0.85; 0.96]	3.8%
Pinto et al., 1993	9	177	0.05	[0.02; 0.09]	3.8%
Suzan et al., 1983	541	1498	0.36	[0.34; 0.39]	3.9%
Tegtmeier et al., 1999	68	72	0.94	[0.86; 0.98]	3.7%
Yavru et al., 2005	137	254	0.54	[0.48; 0.60]	3.8%
Yazici et al., 2019	93	442	0.21	[0.17; 0.25]	3.9%
**Random effects model**		**8927**	**0.62**	**[0.43; 0.79]**	**53.9%**
Heterogeneity: $I^2 = 100\%$, $p = 0$					
**Random effects model**		**13761**	**0.64**	**[0.53; 0.75]**	**100.0%**
**Prediction interval**				**[0.09; 1.00]**	

Heterogeneity: $I^2 = 99\%$, $p = 0$

Test for subgroup differences: $\chi^2_2 = 0.21$, df = 2 ($p = 0.90$)

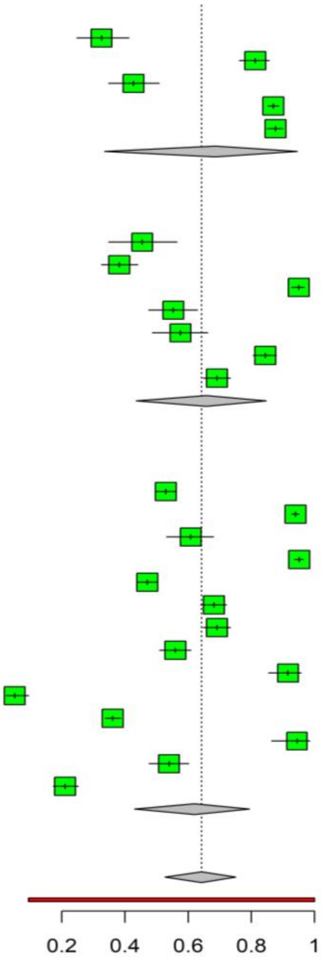

C)

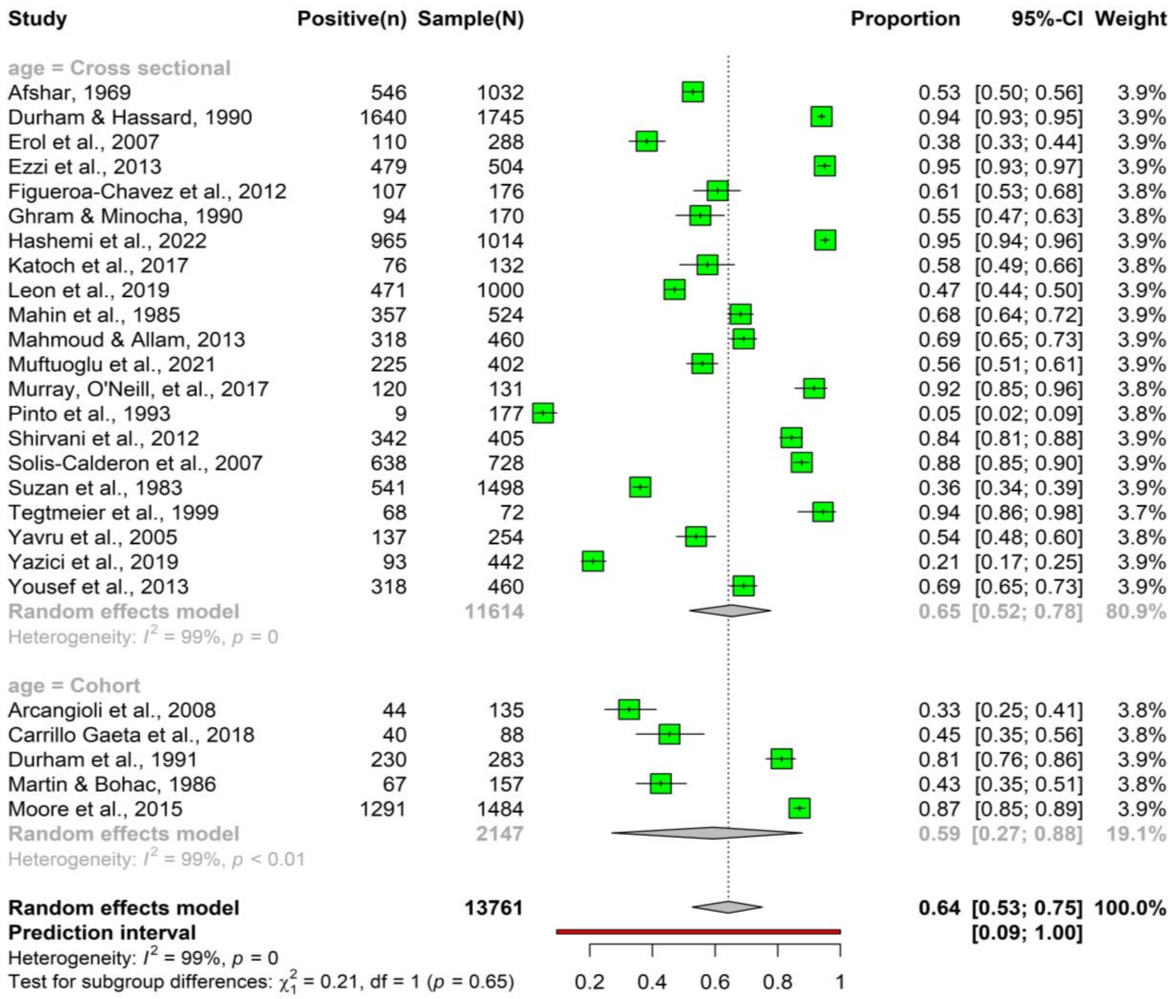

Figure S1. Subgroup analysis of bovine parainfluenza type 3 virus prevalence using antibody detection methods. **A**) age group, **B**) farming system, and **C**) study design.

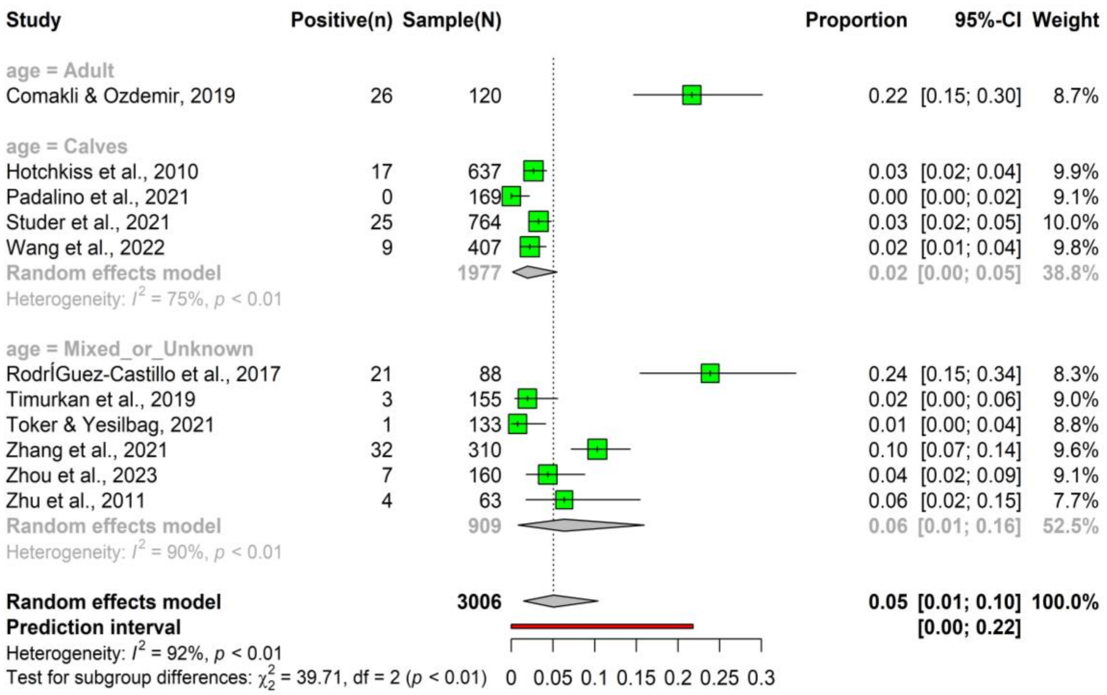
Study Positive(n) Sample(N) Proportion 95%-CI Weight
age = Adult
Comakli & Ozdemir, 2019 26 120 0.22 [0.15; 0.30] 8.7%
age = Calves
Hotchkiss et al., 2010 17 637 0.03 [0.02; 0.04] 9.9%
Padalino et al., 2021 0 169 0.00 [0.00; 0.02] 9.1%
Studer et al., 2021 25 764 0.03 [0.02; 0.05] 10.0%
Wang et al., 2022 9 407 0.02 [0.01; 0.04] 9.8%
Random effects model 1977 0.02 [0.00; 0.05] 38.8%
Heterogeneity: I^2 = 75%, p < 0.01
age = Mixed_or_Unknown
RodrÍGuez-Castillo et al., 2017 21 88 0.24 [0.15; 0.34] 8.3%
Timurkan et al., 2019 3 155 0.02 [0.00; 0.06] 9.0%
Toker & Yesilbag, 2021 1 133 0.01 [0.00; 0.04] 8.8%
Zhang et al., 2021 32 310 0.10 [0.07; 0.14] 9.6%
Zhou et al., 2023 7 160 0.04 [0.02; 0.09] 9.1%
Zhu et al., 2011 4 63 0.06 [0.02; 0.15] 7.7%
Random effects model 909 0.06 [0.01; 0.16] 52.5%
Heterogeneity: I^2 = 90%, p < 0.01
Random effects model 3006 0.05 [0.01; 0.10] 100.0%
Prediction interval [0.00; 0.22]
Heterogeneity: I^2 = 92%, p < 0.01
Test for subgroup differences: χ^2_2 = 39.71, df = 2 (p < 0.01)
0 0.05 0.1 0.15 0.2 0.25 0.3
A)

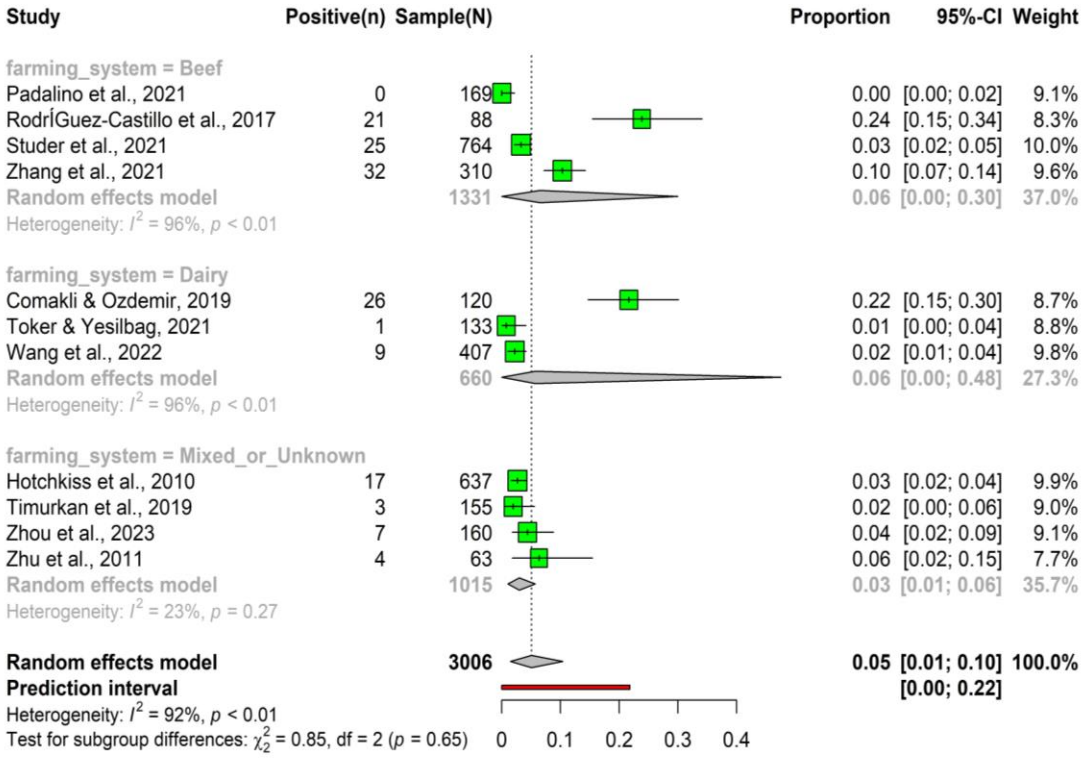
Study Positive(n) Sample(N) Proportion 95%-CI Weight
farming_system = Beef
Padalino et al., 2021 0 169 0.00 [0.00; 0.02] 9.1%
RodrÍGuez-Castillo et al., 2017 21 88 0.24 [0.15; 0.34] 8.3%
Studer et al., 2021 25 764 0.03 [0.02; 0.05] 10.0%
Zhang et al., 2021 32 310 0.10 [0.07; 0.14] 9.6%
Random effects model 1331 0.06 [0.00; 0.30] 37.0%
Heterogeneity: I^2 = 96%, p < 0.01
farming_system = Dairy
Comakli & Ozdemir, 2019 26 120 0.22 [0.15; 0.30] 8.7%
Toker & Yesilbag, 2021 1 133 0.01 [0.00; 0.04] 8.8%
Wang et al., 2022 9 407 0.02 [0.01; 0.04] 9.8%
Random effects model 660 0.06 [0.00; 0.48] 27.3%
Heterogeneity: I^2 = 96%, p < 0.01
farming_system = Mixed_or_Unknown
Hotchkiss et al., 2010 17 637 0.03 [0.02; 0.04] 9.9%
Timurkan et al., 2019 3 155 0.02 [0.00; 0.06] 9.0%
Zhou et al., 2023 7 160 0.04 [0.02; 0.09] 9.1%
Zhu et al., 2011 4 63 0.06 [0.02; 0.15] 7.7%
Random effects model 1015 0.03 [0.01; 0.06] 35.7%
Heterogeneity: I^2 = 23%, p = 0.27
Random effects model 3006 0.05 [0.01; 0.10] 100.0%
Prediction interval [0.00; 0.22]
Heterogeneity: I^2 = 92%, p < 0.01
Test for subgroup differences: χ^2_2 = 0.85, df = 2 (p = 0.65)
0 0.1 0.2 0.3 0.4
B)

C)

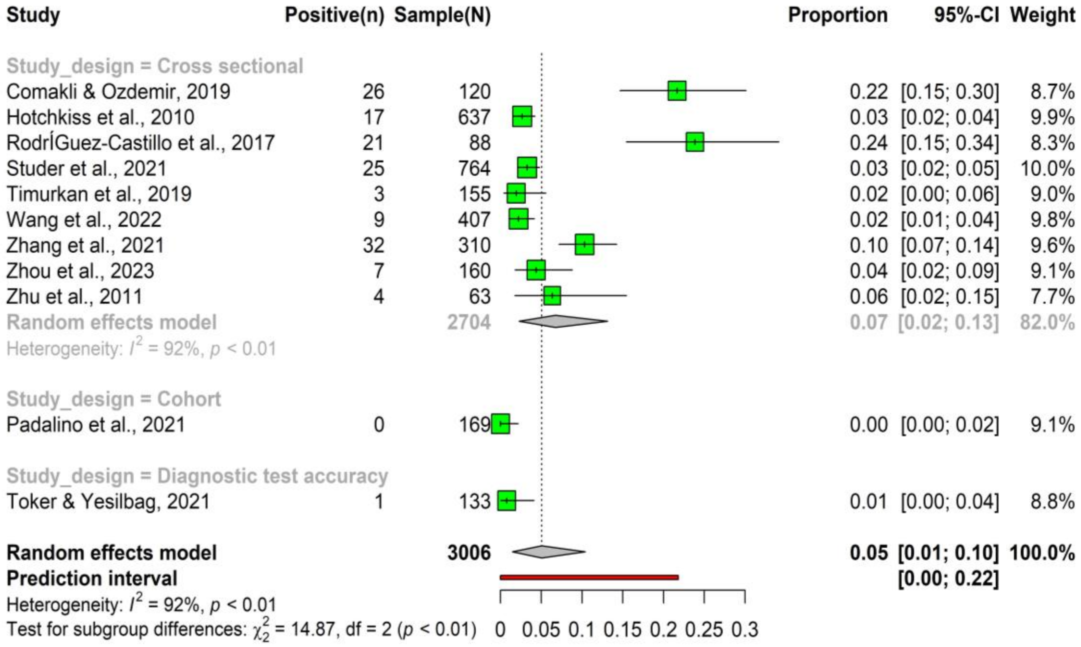

Figure S2. Subgroup analysis of bovine parainfluenza type 3 virus prevalence using nucleic acid detection methods. **A**) age group, **B**) farming system, and **C**) study design.

A)

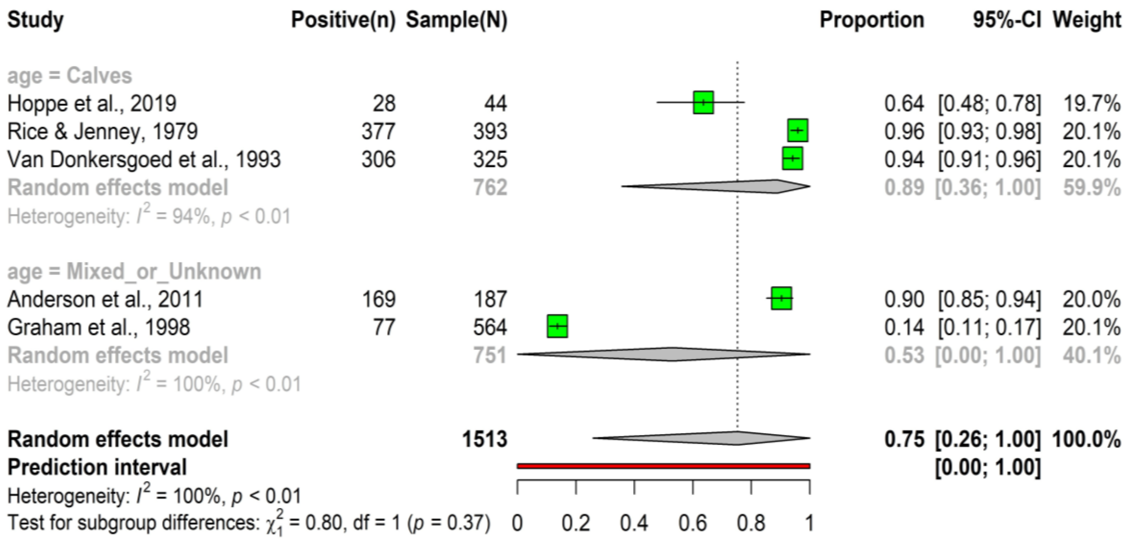

B)

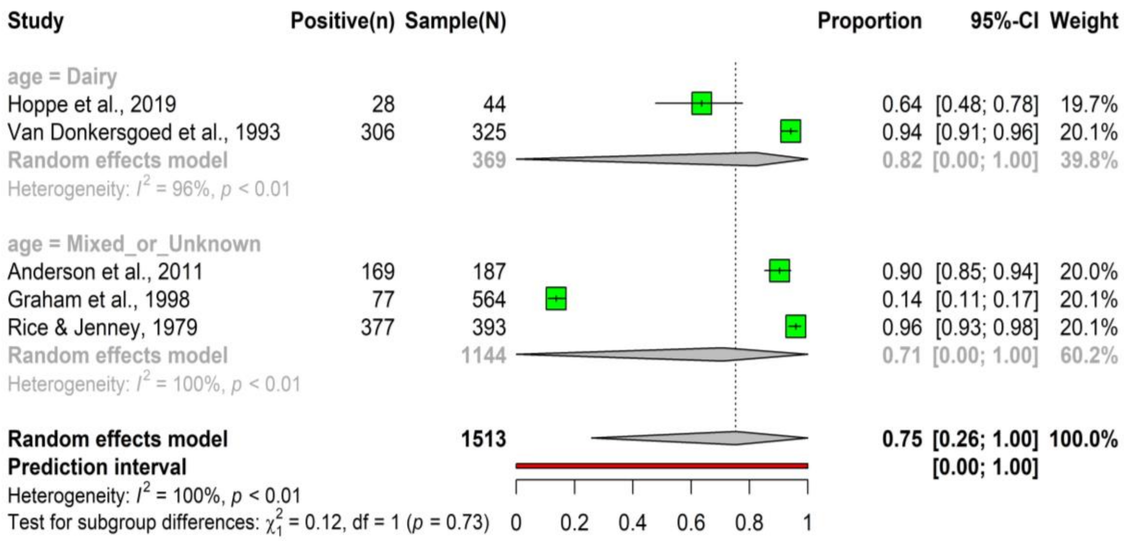

Figure S3. Subgroup analysis of bovine parainfluenza type 3 virus prevalence in cattle with BRDC using antibody detection methods. **A**) age group, and **B**) farming system.

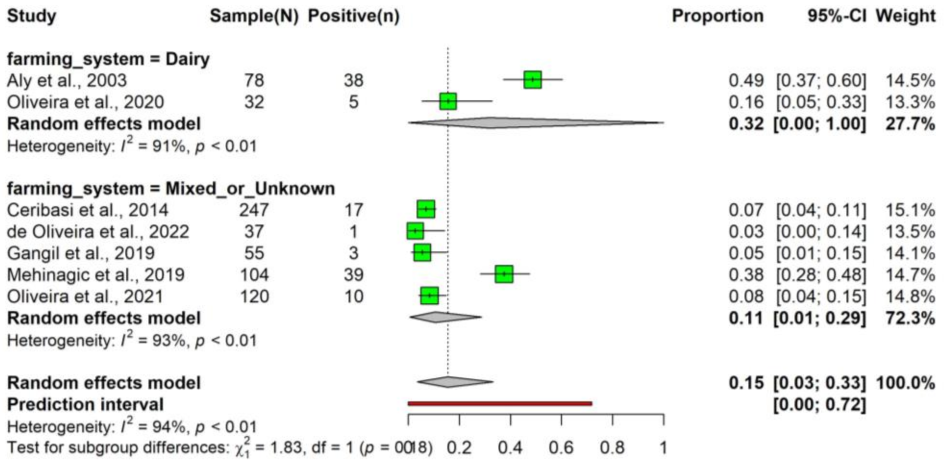

Figure S4. Farming system-based subgroup analysis of bovine parainfluenza type 3 virus prevalence in cattle with BRDC using antigen detection methods.

A)

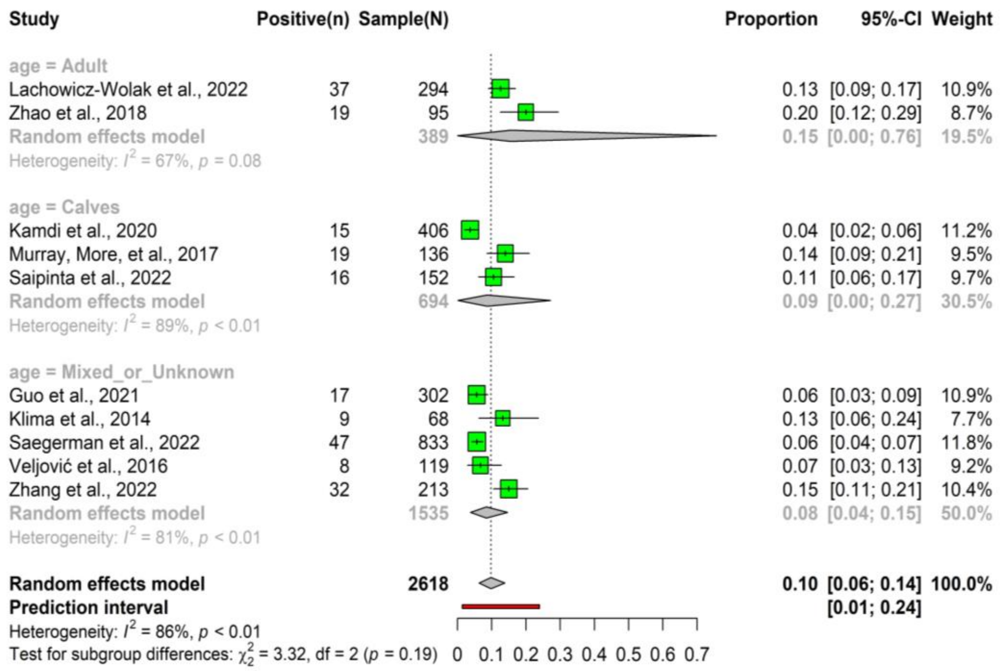
Study
Positive(n)
Sample(N)
Proportion
95%-CI
Weight
age = Adult
Lachowicz-Wolak et al., 2022 37 294 0.13 [0.09; 0.17] 10.9%
Zhao et al., 2018 19 95 0.20 [0.12; 0.29] 8.7%
Random effects model 389 0.15 [0.00; 0.76] 19.5%
Heterogeneity: I2 = 67%, p = 0.08
age = Calves
Kamdi et al., 2020 15 406 0.04 [0.02; 0.06] 11.2%
Murray, More, et al., 2017 19 136 0.14 [0.09; 0.21] 9.5%
Saipinta et al., 2022 16 152 0.11 [0.06; 0.17] 9.7%
Random effects model 694 0.09 [0.00; 0.27] 30.5%
Heterogeneity: I2 = 89%, p < 0.01
age = Mixed_or_Unknown
Guo et al., 2021 17 302 0.06 [0.03; 0.09] 10.9%
Klima et al., 2014 9 68 0.13 [0.06; 0.24] 7.7%
Saegerman et al., 2022 47 833 0.06 [0.04; 0.07] 11.8%
Veljović et al., 2016 8 119 0.07 [0.03; 0.13] 9.2%
Zhang et al., 2022 32 213 0.15 [0.11; 0.21] 10.4%
Random effects model 1535 0.08 [0.04; 0.15] 50.0%
Heterogeneity: I2 = 81%, p < 0.01
Random effects model 2618 0.10 [0.06; 0.14] 100.0%
Prediction interval [0.01; 0.24]
Heterogeneity: I2 = 86%, p < 0.01
Test for subgroup differences: χ22 = 3.32, df = 2 (p = 0.19)
0 0.1 0.2 0.3 0.4 0.5 0.6 0.7

B)

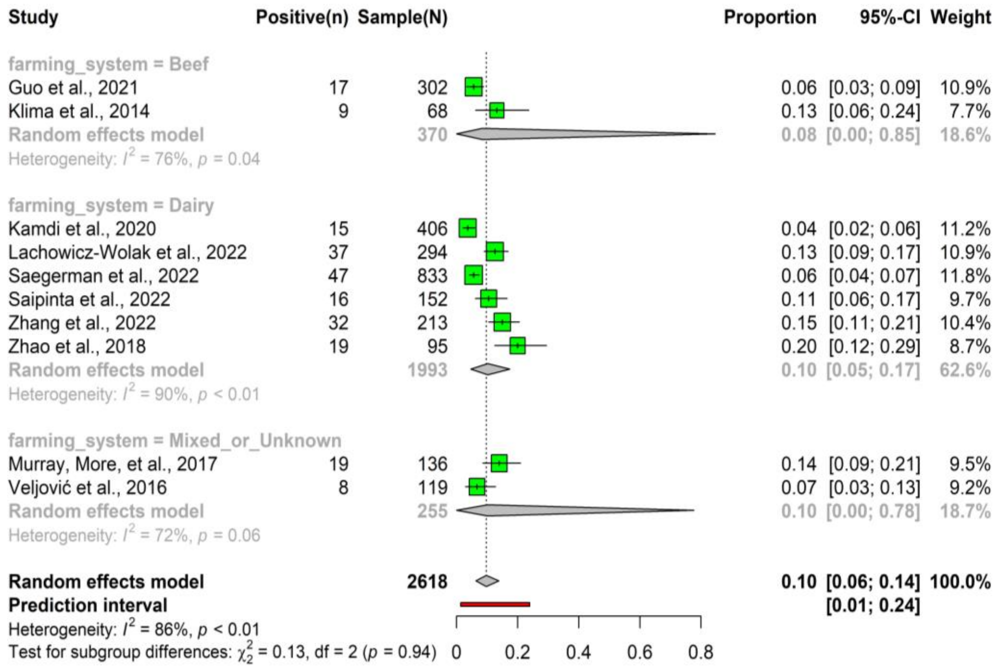
Study
Positive(n)
Sample(N)
Proportion
95%-CI
Weight
farming_system = Beef
Guo et al., 2021 17 302 0.06 [0.03; 0.09] 10.9%
Klima et al., 2014 9 68 0.13 [0.06; 0.24] 7.7%
Random effects model 370 0.08 [0.00; 0.85] 18.6%
Heterogeneity: I2 = 76%, p = 0.04
farming_system = Dairy
Kamdi et al., 2020 15 406 0.04 [0.02; 0.06] 11.2%
Lachowicz-Wolak et al., 2022 37 294 0.13 [0.09; 0.17] 10.9%
Saegerman et al., 2022 47 833 0.06 [0.04; 0.07] 11.8%
Saipinta et al., 2022 16 152 0.11 [0.06; 0.17] 9.7%
Zhang et al., 2022 32 213 0.15 [0.11; 0.21] 10.4%
Zhao et al., 2018 19 95 0.20 [0.12; 0.29] 8.7%
Random effects model 1993 0.10 [0.05; 0.17] 62.6%
Heterogeneity: I2 = 90%, p < 0.01
farming_system = Mixed_or_Unknown
Murray, More, et al., 2017 19 136 0.14 [0.09; 0.21] 9.5%
Veljović et al., 2016 8 119 0.07 [0.03; 0.13] 9.2%
Random effects model 255 0.10 [0.00; 0.78] 18.7%
Heterogeneity: I2 = 72%, p = 0.06
Random effects model 2618 0.10 [0.06; 0.14] 100.0%
Prediction interval [0.01; 0.24]
Heterogeneity: I2 = 86%, p < 0.01
Test for subgroup differences: χ22 = 0.13, df = 2 (p = 0.94)
0 0.2 0.4 0.6 0.8

C)

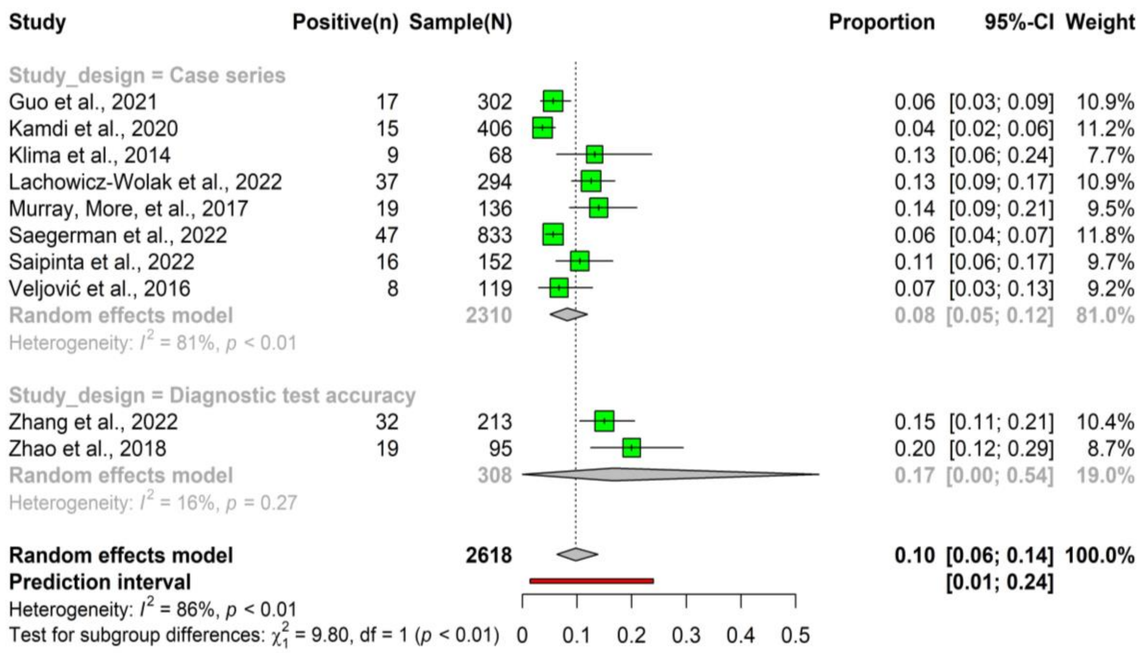

Figure S5. Subgroup analysis of bovine parainfluenza type 3 virus prevalence in cattle with BRDC using nucleic acid detection methods. **A**) age group, **B**) farming system, and **C**) study design.
